# The cross-reactivity of the enterovirus 71 to human brain tissue and identification of the cross-reactivity related fragments

**DOI:** 10.1186/1743-422X-7-47

**Published:** 2010-02-22

**Authors:** Chun Shi Jia, Jiang Ning Liu, Wan Bo Li, Chun Mei Ma, Shu Zhu Lin, Yi Hao, Xue Zhong Gao, Xiao Lin Liu, Yan Feng Xu, Lian Feng Zhang, Chuan Qin

**Affiliations:** 1 Key Laboratory of Human Diseases Comparative Medicine, Ministry of Health, Institute of Laboratory Animal Science, Chinese Academy of Medical Sciences (CAMS) & Comparative Medicine Centre, Peking Union Medical Collage (PUMC), Chao Yang Strict, Pan Jia Yuan Nan Li No.5, Beijing 100021, PR China; 2 Fuyang People's Hospital, Lu Ci Street No.63, Fu Yang city, An Hui province 236003, PR China

## Abstract

**Background:**

EV71 occasionally cause a series of severe neurological symptoms, including aseptic meningitis, encephalitis, and poliomyelitis-like paralysis. However, the neurological destruction mechanism was remained to be clarified. This study described the cross reaction between EV71 induced IgG and human brain tissue.

**Results:**

Cross reaction of the IgG from 30 EV71 infected patients' sera to human tissues of cerebra was observed, which suggested that some EV71 antigens could induce IgG cross-reactivity to human cerebra. To identify the regions of EV71 virus that containing above antigens, the polypeptide of virus was divided into 19 peptides by expression in prokaryotes cell. Mouse anti-sera of these peptides was prepared and applied in immunohistochemical staining with human adult and fetus brain tissue, respectively. The result indicated the 19 peptides can be classified into three groups: strong cross-reactivity, weak cross-reactivity and no cross-reactivity with human brain tissue according the cross reaction activity. Then, the increased Blood Brain Barrier (BBB) permeability and permits IgG entry in neonatal mice after EV71 infection was determined.

**Conclusion:**

EV71 induced IgG could enter BBB and cross-reacted with brain tissue in EV71 infected neonatal mice, and then the peptides of EV71 that could induce cross-reactivity with brain tissue were identified, which should be avoided in future vaccine designing.

## Background

Human enterovirus (EV71) was first described by Schimdit et al. in 1974 [1], which belonging to Picornaviridea family and has a single positive stranded ribonucleic acid (RNA) of about 7,500 nucleotides [2,3]. There have 13 large and small reported outbreaks of EV71 throughout the world since then, which main leads to high prevalence of hand, foot and mouth disease (HFMD) in infants and children under 6 years old [4-6]. In past decades, countries in the Asia-Pacific region have experienced an increased occurrence of EV71 associated HMFD outbreak [7-11]. Most of EV71 infection are benign and self-limited in nature, however, EV71 infection has been reported to cause neurological disease manifesting as aseptic meningitis, encephalitis or poliomyelitis-like acute flaccid paralysis, and neurological originated pulmonary edema or hemorrhage was the main reason of lethal symptom [4,12]. The central nervous system (CNS) injury dependent EV71 neuropathology is supposed as the main reason kills neuron and then lead to subsequent neurological destruction [4,13-18]. Although a certain numbers of research work have been carried out, as no abundant virus titer was detected in the CNS during EV71 challenge in mice model, meanwhile, the attenuated EV71 strain can still induce weak neurological symptoms in monkey, the detailed mechanism of CNS dysfunction is remained to be clarified [15,19].

EV71 virus infection was reported to increase the permeability of BBB [20,21]. However, as enter of virus into cranial was dependent on a retrograde axonal neuronal transmission way, the increased permeability in BBB was presumed not essential for virus through BBB. As described in Epilepsy, self immunity caused by the common antigens between virus and cell receptors lead to neuron injury, in which the central nervous system (CNS) is attacked by the immune system and that provide a inspiration for the possible new way during the pathology of EV71 infection study [22].

In current study, the sera isolated from EV71 infected patients were indicated to cross reaction with the human tissues of cerebrum by immunohistochemical staining and then the regions can elicit cross-reactivity with normal brain tissues were identified.

## Results

### Cross reaction of the IgG from EV71 infected patients' sera to human tissues of cerebra

**Figure 1:**
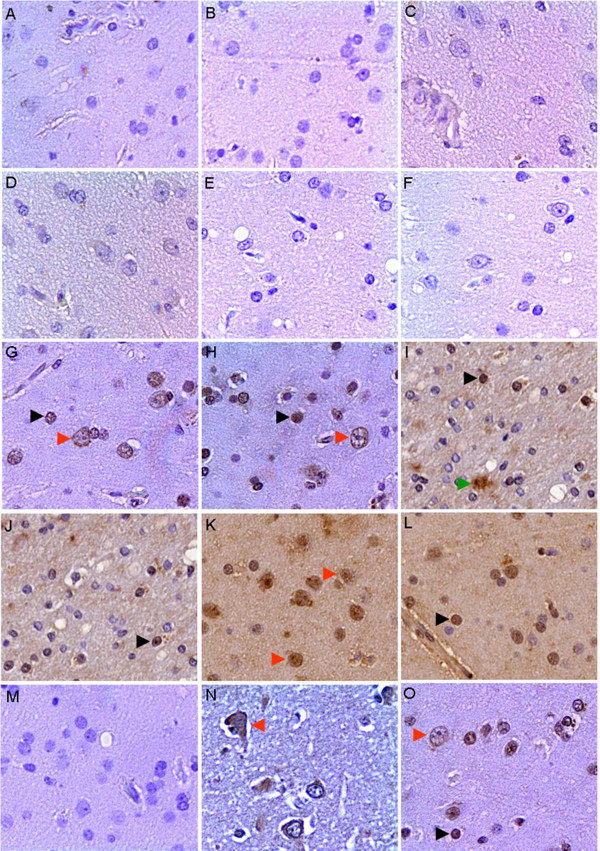
**Cross reaction of the IgG from EV71 infected patients' sera with human tissues of cerebra**. The serum was replaced by PBS buffer as blank control (a), and naive sera from five health donors were used as negative control (b-f). The sera of CNILASTB-16 (g), CNILASTB-19 (h), CNILASTB-1 (i), CNILASTB-2 (j), CNILASTB-4 (k) and CNILASTB-9 (l) were used as primary antibodies in immunohistochemical staining with the cerebra of adult. The IgG fraction in the sera of NORMAL-1 (m), CNILASTB-12 (n) and CNILASTB-4 (o) were used as primary antibodies in immunohistochemical staining with the cerebra of adult to exclude the interference of the remained ingredients in sera. The stained glial cell, stroma and neuron were denoted with black, green and red arrows respectively (×200).

**Figure 2:**
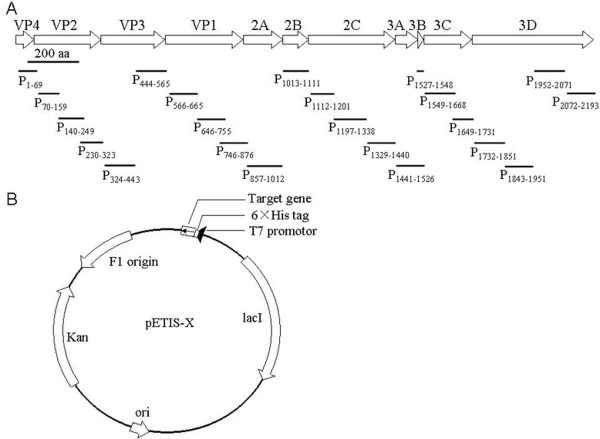
**Diagrams of divided 22 peptides from EV71 and expression construction**. The position and length of 22 peptides divided from EV71 (a) and expression constructs for 22 peptides (b) were diagramed.

A large outbreak of HFMD in infants and children was happened in Fuyang region of China in the spring of 2008 [23,24]. Thirty sera from children with HFMD was collected, who was infected with EV71 after RT-PCR diagnosis the specimen of throat swab, and the presence of EV71 induced antibody (both IgM and IgG) in all the thirty sera was verified by ELISA (data not show). As neurological pulmonary edema or hemorrhage was the main reason of lethal symptom [25,26], and our inactivated virus vaccine showed neurological virulence while applied in primates test (date not show), the 30 sera from EV71 infected patients were used as primary anti-sera to perform the immunohistochemical staining with adult human tissues of cerebra (Fig. 1). The normal sera from five donors (four children and one adult) were used as negative control, in which the anti- EV71 IgG and IgM was free with ELISA analysis. The human tissues of cerebra was not stained obviously with negative control sera (Fig. 1B-F), while the human tissues of cerebra was stained on the neuron glial cell, neuron and stroma by the patients sera (Fig. 1G-L). The positive staining was observed in all of the 30 sera from EV71 infected patients and 86% of the 30 sera showed cross reaction with 10-40% stained cells (Table 1). To exclude the interference of remained ingredients in sera, the IgG fraction in three sera samples were purified and used as primary antibodies in immunohistochemical staining with human brain tissues, and the results were consistent to sera's experiment in neuron glial cell and neuron, but not in stroma (Fig. 1M-O). These results indicated the presence of specific IgG in the EV71 infected patient sera having the cross-reactivity activity to human cerebra and suggested that some EV71 antigens could induce cross-reactivity to human cerebra. The expression and purification of the peptides disassembled from EV71To identify the regions of EV71 virus that can induce antibodies binding with human brain tissue, the genome of EV71 was divided into 22 regions in sequence, which encode peptides between 22~156 amino acids (Fig. 2A), and the nearby fragments in a functional gene have 12 to 39 bp overlap to avoid the miss of epitope. EV71 of Fuyang-0805 strain isolated from Anhui province of China was used as the templates for primers designing and RT-PCR (see Additional file 1). Targeted cDNA fragments were cloned into pETIS vector modified from pET28a (+) (Fig. 2B), and then the fusion peptides with six histidine residues tag were over-expressed. Nineteen of the 22 targeted cDNA fragments were synthesized in insoluble inclusion body forms by *E. coli*, but three of them, P_1013-1111_, P_1112-1201 _and P_1527-1548_, were failed to expression in this bacteria strain. After Ni-HTA purification, all of the 19 peptides were purified to a purity of 95% (see Additional file 2).

**Table 1:** Evaluation the immunohistochemical staining results of patients' sera to adult human cerebra

Sera no/human sex/age	Clinical features/Diagnosis	Day of sera collection after onset	Evaluation score
NORMAL-1/F/1.5	NO	-	-
NORMAL-2/M/3.5	NO	-	-
NORMAL-3/F//4	NO	-	-
NORMAL-4/M/2.6	NO	-	-
NORMAL-5/F/23	NO	-	-
CNILASTB-1/M/1.5	Fever, HMFD/Brain stem encephalitis	1	+ + +
CNILASTB-2/F/2.8	Fever, irritability, myoclonus, HFMS/Brain stem encephalitis	1	+ +
CNILASTB-3/M/3.6	Fever, myoclonus, HMFD/Brain stem encephalitis	1	+ + +
CNILASTB-4/F/4.5	Fever, irritability, HMFD/Brain stem encephalitis	3	+ + + +
CNILASTB-5/M/2.5	HMFD/-	0	+ +
CNILASTB-6/M/1.8	HMFD/-	0	+ +
CNILASTB-7/M/1.5	Fever, vomiting, irritability, HMFD/Brain stem encephalitis	0	+ + +
CNILASTB-8/F/2.4	HMFD/-	0	+ +
CNILASTB-9/F/2.5	Fever, vomiting, motor weakness/Aseptic meningitis	3	+ + + +
CNILASTB-10/M/1.5	Fever, vomiting, HMFD/Brain stem encephalitis	1	+ + +
CNILASTB-11/M/2.2	Fever, irritability, myoclonus, HMFD/Brain stem encephalitis	2	+ + +
CNILASTB-12/M/1.6	HMFD/-	0	+
CNILASTB-13/M/1.2	HMFD/-	0	+ +
CNILASTB-14/M/1.8	Fever, irritability,/Brain stem encephalitis	0	+ +
CNILASTB-15/F/2.6	Fever, lethargy, ataxia, HFMS/Brain stem encephalitis, neurogenic shock, pulmonary edema	0	+ + + +
CNILASTB-16/M/4.6	HFMD/-	0	+
CNILASTB-17/M/5.4	HFMD/-	1	+ +
CNILASTB-18/F/3.2	Fever, vomiting, lethargy, motor weakness/Aseptic meningitis	1	+ + +
CNILASTB-19/F/5.2	HFMD/-	2	+
CNILASTB-20/F/5	Fever, vomiting, lethargy, nystagmus, shock/Brain stem encephalitis, pulmonary edema	1	+ + +
CNILASTB-21/M/3.6	Fever, vomiting, headache/Aseptic meningitis	2	+ + + +
CNILASTB-22/F/3.2	HFMD/-	0	+
CNILASTB-23/F/2.4	Irritability, lethargy, apathy, myoclonus, HFMS/Aseptic meningitis	2	+ + +
CNILASTB-24/M/1.7	Fever, vomiting, lethargy, nystagmus, myoclonus, shock/Brain stem encephalitis, neurogenic shock, pulmonary edema	2	+ + +
CNILASTB-25/M/3.4	HFMD/-	1	+ +
CNILASTB-26/F/4.6	HFMD/-	1	+ +
CNILASTB-27/F/2.8	Fever, vomiting, headache, irritability, HMFD/Aseptic meningitis	1	+ +
CNILASTB-28/M/3	Fever, lethargy, myoclonus, shock/Aseptic meningitis	2	+ + +
CNILASTB-29/F/2	HFMD/-	2	+ +
CNILASTB-30/M/3.2	Fever, vomiting, headache, myoclonus, HFMS/Brain stem encephalitis	1	+ + + +

- No obvious staining was observed;

+ 0~10% cell was stained;

+ + 11%~20% cell was stained;

+ + + 21%~30% cell was stained;

+ + + + 31%~40% cell was stained.

### Identification of EV71 fragments inducing cross-reactivity to human brain tissue

**Figure 3:**
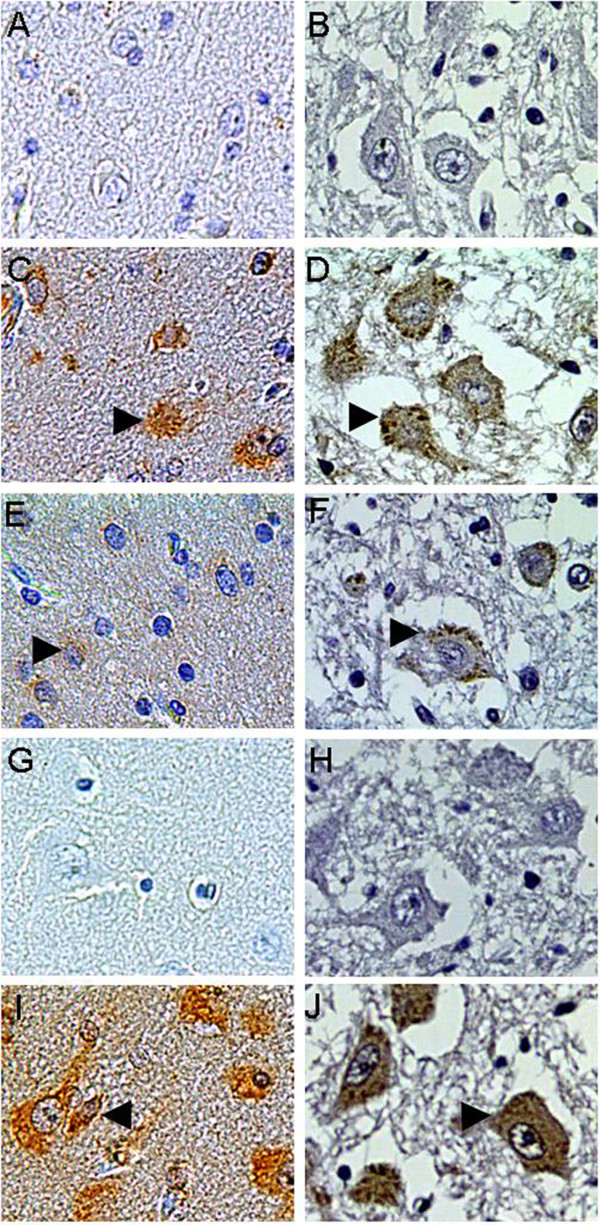
**Identification of EV71 fragments inducing cross immunity to human brain tissue by immunohistochemical staining**. Left panel was adult human cerebra and the right panel was human fetus medulla. The naive mice sera was used as negative control (a & b). The serum of immunized mice with peptide of P_646-755 _was one of the four strong cross immunity peptides(c & d). P_2072-2193 _was one of the ten weak cross immunity peptides (e & f). P_140-249 _was one of the five no cross immunity peptides (g & h). The serum of immunized mice with heat inactivated virus was used as positive control (i & j). The staining sites were denoted with arrows (×200).

The 19 purified peptides were applied to immunize the 6 weeks female ICR mice according polyclonal antibody preparation procedure described in the Method. The sera of immunized mice were collected, and the titers of IgG against EV71 in the sera were determined by ELISA respectively (Table 2). No lesions in any of the brain tissues of immunized mice were observed (data not show), then, the 19 sera were applied in immunohistochemical staining to identify which sera have cross reaction with human adult and fetus brain tissues, respectively (Fig. 3). The anti-sera of P_230-323_, P_646-755_, P_857-1012 _and P_1329-1440 _showed strong staining with neuron plasma in both adult human cerebra and fetus medulla compared with the negative control (Fig. 3A-D). The anti-sera of P_1-69_, P_324-443_, P_444-565_, P_566-665_, P_746-876_, P_1441-1526_, P_1549-1668_, P_1732-1851_, P_1952-2071_and P_2072-2193 _showed weaker staining with the human brain tissues than the anti-sera of P_230-323_, P_646-755_, P_857-1012 _and P_1329-1440 _(Fig. 3E and 3F). The anti-sera of P_70-159_, P_140-249_, P_1197-1338_, P_1649-1731_, P_1843-1951 _did not show staining with both the adult human cerebra and fetus medulla sections (Fig. 3G and 3H). This result indicated that the peptides of P_230-323_, P_646-755_, P_857-1012 _and P_1329-1440 _could induce strong IgG cross-reactivity to human brain tissue. The significant of cross reactivity was not relevant to the specific IgG titer induced by individual peptides (Table 2), which indicated the cross reactivity was a specific IgG behavior rather than an antibody dose dependent artifact.

**Table 2:** ELISA determines the titers of sera from immunized mice to peptides and virus

	OD_450 _Value	
	
Peptide name	**Peptides**^a^	**Virus**^b^
P_1-69_	1.231 ± 0.114	0.231 ± 0.034
P_70-159_	1.781 ± 0.221	0.446 ± 0.041
P_140-249_	1.940 ± 0.234	0.557 ± 0.071
P_230-323_	1.126 ± 0.167	0.359 ± 0.053
P_324-443_	1.125 ± 0.138	0.678 ± 0.072
P_444-565_	1.864 ± 0.245	1.246 ± 0.116
P_566-665_	2.142 ± 0.175	1.648 ± 0.157
P_646-755_	0.948 ± 0.056	0.467 ± 0.067
P_746-876_	1.084 ± 0.097	0.647 ± 0.064
P_857-1012_	1.562 ± 0.149	0.169 ± 0.022
P_1197-1338_	2.214 ± 0.195	0.328 ± 0.016
P_1329-1440_	1.194 ± 0.134	0.294 ± 0.008
P_1441-1526_	1.162 ± 0.158	0.175 ± 0.006
P_1549-1668_	0.955 ± 0.037	0.186 ± 0.011
P_1649-1731_	1.556 ± 0.115	0.268 ± 0.018
P_1732-1851_	1.678 ± 0.160	0.327 ± 0.020
P_1843-1951_	1.966 ± 0.138	0.267 ± 0.013
P_1952-2071_	1.763 ± 0.129	0.291 ± 0.026
P_2072-2193_	1.567 ± 0.146	0.488 ± 0.038

^a ^the sera were used to determine the titer to its self peptide in a dilution 1:50,000.

^b^ the sera were used to determine the titer to virus in a dilution 1:1,000. Values represent means ± SD of three independent experiments.

### EV71 infection increased BBB permeability and IgG transport

**Figure 4:**
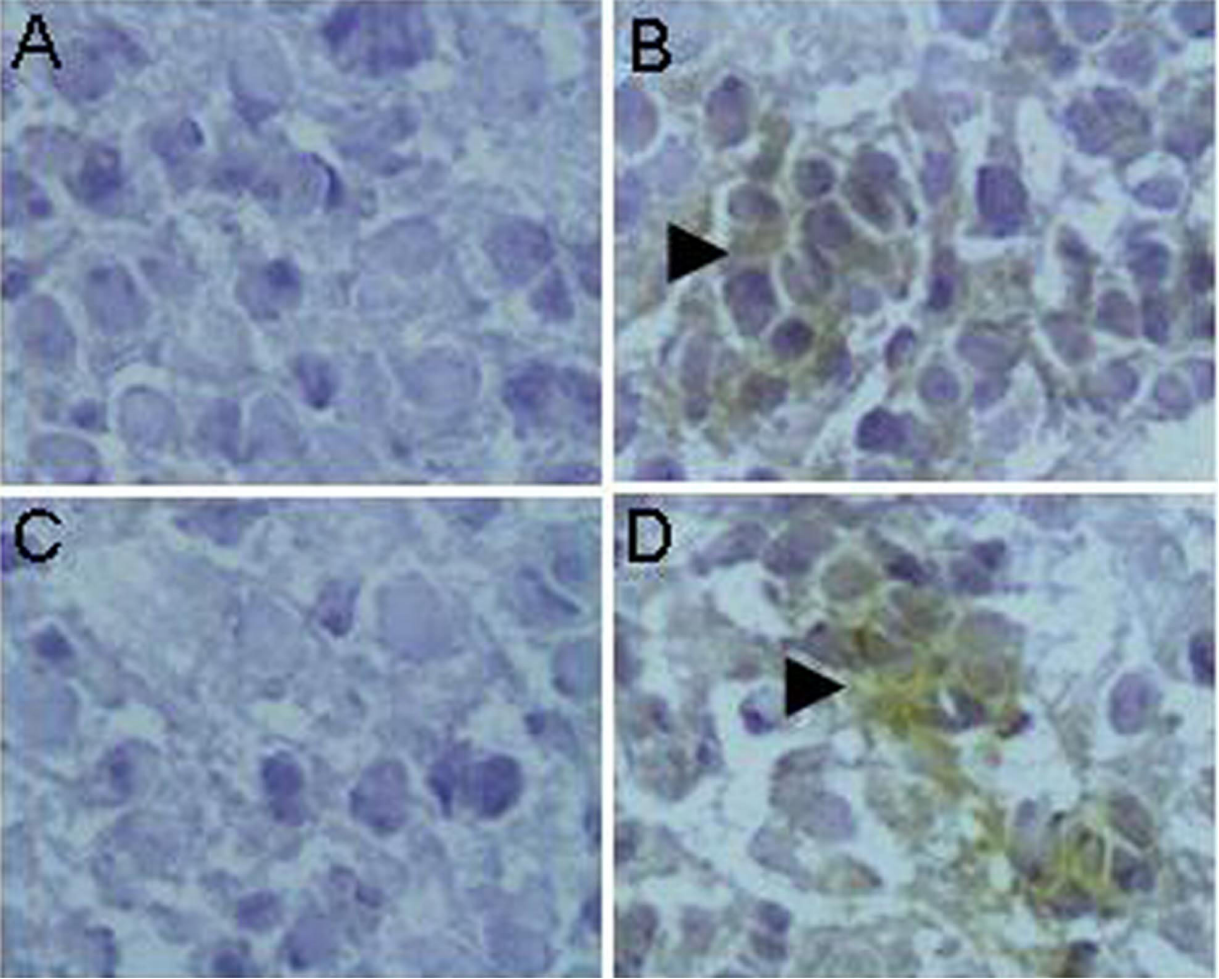
**Detection the intracranial IgG in mice after intravenous injection**. The normal neonatal mice injected with naive IgG. (a), EV71 infected neonatal mice injected with mice naïve IgG. (b), normal neonatal mice injected with EV71 induced IgG. (c) and EV71 infected neonatal mice injected with EV71 induced IgG.(d) were compared by the immunohistochemical test. The staining sites were denoted with arrows (× 200).

An EV71 mice infection model was build in our laboratory (unpublished data), neonatal mice were intraperitoneal infected with 5 × 10^5 ^TCID_50 _virus within 24 h after birth, the virus replication was detected in brain, lung, small intestine and skeletal muscle at 5 dpi by Real-time PCR and virus titer determination in RD cell, respectively. Then, the virus locations in these tissues were verified by immunohistochemical staining with a monoclonal antibody of EV71 (Millipore). Large area of neuron apoptosis was observed in cerebrum and medulla of brain tissue. In small intestine, intestinal villus interstitial edema and epithelial cell vacuolar degeneration were observed. In skeletal muscle, inflammation, muscle fiber degeneration and necrosis were scarcely observed. However, although virus replication and virus antigens were detected in lung, no obvious lesion was observed in this organ. As no lesions in all the brain tissues of immunized adult mice were observed, we studied the BBB permeability of neonatal mice upon EV71 infection. Naive or EV71 induced IgG was intravenous injected into the neonatal mice upon EV71 infection or not, respectively. And the detection results were shown in Fig. 4, both weak staining sites were observed in EV71 infected mice brain tissues after naive or EV71 induced IgG injection (Fig. 4B and 4D), while the staining was scarcely observed in normal mice brain tissue after IgG injection (Fig. 4A and 4C). And the intracranial IgG showed a weak diffused distribution in the brain tissue of EV71 infected mice, which indicated that compared to normal neonatal mice, the BBB permeability of EV71 infected mice was increased and permits both the naïve IgG and EV71 induced IgG entry.

## Discussion

The HFMD occasionally causes a series of severe neurological symptoms, including aseptic meningitis, encephalitis, poliomyelitis-like paralysis and neurological originated pulmonary edema or hemorrhage [25,27-29], especially the latter was the main reason of lethal symptom in infants and children [4,12]. Previous study suggested the neuropathological symptom was caused by the EV71 infection in CNS [30-34]. However, Chen CS et al. reported that although the infection led to a persistent viremia and a transient increase in BBB permeability, but only low levels of virus could be detected in the mice brain [15], and Arita M et al. reported that the monkeys vaccinated with an attenuated EV71 showed the broad range of CNS tissues inflammation related to the peak stages of IgM and IgG producing, but without the efficient virus replication [19]. Those results suggested that the pathogenesis of neurological symptoms with HFMD may have more mechanisms.

We find that all of the tested EV71 infected patients' sera were presence of IgG to cross-react with health human brain tissues (Fig. 1), which suggested that a potential cross-reactivity of EV71 with human brain tissues. To identify the fragments of EV71 that induce cross-reactivity to human brain tissue, the whole genome of virus was divided into 22 fragments sequentially. The 19 of 22 fragments were successfully expressed and purified, however, 3 fragments was failed to expressed in prokaryote environment and those were given up. For cross-reactivity assay, the polyclonal mouse anti-sera against the 19 peptides were prepared and applied in the immunohistochemical analysis with the adult human cerebra and fetus medulla. The results indicated the 19 peptides can be divided into three groups according the cross reaction activity. There were 4 peptides showed strong cross-reactivity with human brain tissues. There were 10 peptides showed weak cross-reactivity with human brain tissues and there were 5 peptides showed no cross-reactivity with human brain tissues. Sera of patients and the strong cross-reactivity peptides showed a similar pattern on the staining of neuron of the human brain tissues (Fig. 1 and 3). These suggested the strong cross-reactivity peptides were potential common antigens of the EV71 with human brain tissue, however, the brain binding partner of EV71 induced IgG was not identified in this work. The peptides that elicit antibodies can bind to human brain tissue was dispersed in whole proteome of EV71, rather than gathered in one or several structural proteins. So, the potentially self-reaction antibodies would be induced over the whole process of virus infection including virus invasion, propagation and releasing. In previous study, both attenuated and avirulent virus or virus like particles were able to induce significant neutralization antibody, relax the clinical symptoms and reduce mortality rate of laboratory animals upon lethal virus challenge[19,35,36]. As indicated in cynomolgus monkeys, although an attenuated EV71 strain showed obvious protection activity in *vivo*, the inoculated monkeys still manifested weak neurological symptom[19]. VP1, located in the surface of virus particles, was thought as the predominant epitope clustering protein, has been broadly used as subunit vaccine to substitute whole virus and elicit significant protection on neonatal animals [37-42]. However, the IgG induced by P_646-755_, which belongs to VP1, elicited strong cross-reactivity to human brain tissue, so the neurological safety of whole virus or VP1 protein as vaccine should be concerned before clinical application. Many disease and virus infection, especially the neurological disease can increase the BBB permeability, e.g. stroke, human immunodeficiency virus, Alzheimer's disease, brain cancer, and bacterial infections of the CNS [20,21,43], likewise, the increased BBB permeability after EV71 infection was verified in a mice model [15]. Our result also indicated the increase of BBB permeability after EV71 infection in infant mice and further more, the increase of BBB permeability after EV71 infection could result in the entrance and localization of the IgG into brain tissues. Autoimmune disease was reported in many previous studies, such as celiac disease, sclerosis, encephalitis, Diabetes mellitus [44-48]. As the BBB in infant can be destructed upon EV71 infection[15] and the EV71 can induce cross-reactivity IgG, therefore, whether the intracranial entry of EV71 elicited IgG is one of the mechanisms of neurological pathogeneses in HFMD patients was remained to be clarified in further study. However, the cross reaction fragments of the viruses should be concerned in future vaccine designing.

## Conclusion

In conclusion, this study initially showed the cross-reactivity between EV71 induced IgG and human brain tissue, and the peptides of EV71 that can bring IgG based cross reaction was identified. We observed the increase of BBB permeability in neonatal mice under EV71 infection and the entry of brain cross reactivity IgG, which was supposed play a role in subsequent clinical symptoms.

## Material and methods

### Cell and virus

RD cells (human rhabdomyosarcoma) were maintained in Dulbecco's modified Eagle's medium with 10% fetal bovine serum [49]. EV71 FuYang stock virus strain (Fuyang-0805), which belongs to C4, the predominant genotype in recent outbreaks in Asia (GenBank accession number EU703812) was grown in RD cells as described by He YQ et al. and Lin JY et al [50,51].

The infected cell culture was disrupted by three freeze-thaw cycles, then the cell debris was removed by centrifuged at 3,000 g for 20 min, the virus was collected by centrifuged at 80,000 × g for 3 h, and then was ected was pur normal neuron resuspended in phosphate buffered saline (PBS) [36] to prepare the working stocks containing 10^8 ^TCID_50_/ml [13].

### Clone of the EV71 DNA fragments and construction of the expression constructs

The EV71 genomic RNA was extracted from the culture fluid of infected cells using a High-pure viral RNA purification kit (Qiagen). Reverse transcription-PCR (RT-PCR) was performed using a ReverTra-Plus kit (Invitrogen) to clone the full length cDNA [19]. The EV71 cDNA was used as template for peptide coding regions amplification. The primers and associated restriction enzyme sites were subjected in Additional file 1. The cloned fragments were inserted into cloning sites of pETIS vector under the T7 promoter and His tag was added at the N-terminal of the target peptide to form a fusion peptide with His tag. The expression plasmids were verified by sequencing analysis respectively.

### Peptide expression and purification

*Escherichia. coli *BL21 (DE3) was transformed by the constructed expression plasmids for protein expression. The DE3 bacteria strain was cultured with Luria-Bertani (LB) medium with 50 μg/ml kanamycin at 37°C, 200 rpm. After the OD_600 _of culture up to 0.6, the IPTG was added to a final concentration of 0.5 mM into the medium and cultured for 8 h at 25. The bacteria pellet was harvested by centrifugation at 4, 000 × g for 20 min, and the expression of targeted proteins were detected by SDS-PAGE.

Protein purification was performed according to the protocol of Novagen with Ni-HTA resin. Briefly, the bacteria pellet was resuspended in buffer B (8 M urine, 0.1 M sodium phosphate salt, 0.01 M Tris-HCl, pH = 8.0) and incubated at 37°C for 30 min, the cell debris was removed by centrifuged at 20,000 × g for 20 min. Then the supernatant was loaded onto a Ni-HTA resin column. The column was washed with 10 fold column volumes of washing buffer C (8 M urine, 0.1M sodium phosphate salt, 0.01 M Tris-HCl, pH = 6.3). The target protein was then eluted with buffer D (8 M urine, 0.1 M sodium phosphate salt, 0.01 M Tris-HCl, pH = 4.5) and dialyzed against 0.9% NaCl, and then the purity of peptides were detected by SDS-PAGE. The concentration of protein was measured by Bradford method [52].

### Immunity

ICR mice were provided by the Institute of Laboratory Animal Science, Peking Union Medical College. All the mice were bred in an AAALAC-accredited facility and the use of animals was approved by the Animal Care and Use Committee of the Institute of Laboratory Animal Science of Peking Union Medical College (GC09012). The adult mice were used for polyclonal antibody preparation. The peptides were dissolved as 1.0mg/ml of each peptide in 0.9% NaCl and then formulated with same volume of Freund complete adjuvant (Sigma) according to the manufacturer's instructions. The injected dose of the peptides was 100 μl/mouse given through intraperitoneal injection (i.p.). The heat inactivated EV71 were dissolved as 1.0 × 10^9 ^TCID_50_/ml in 0.9% NaCl and the injected dose of the virus was 100 μl per mouse. One week after the first injection, the animals were boosted at the same dose of peptides or the heat inactivated virus formulated with same volume of Freund incomplete adjuvant (Sigma) through intraperitoneal injection(i.p.) and the mice were reboosted weekly for 2 times.

### ELISA

The levels of specific IgG against EV71 or peptides from the immunized mice or EV71 infected patients were determined by enzyme-linked immunosorbent assay (ELISA). Briefly, microtiter plates were coated with 100 μl of heat inactivated virus (1.0 × 10^8 ^TCID_50_) or peptides (10 μg/ml) in carbonate coating buffer (15 mM Na_2_CO_3_, 35 mM NaHCO_3_, pH 9.6). The plates were incubated at 4°C overnight and then incubated with 1% BSA in PBS for 2 h at room temperature to prevent non-specific binding, serial dilutions of test sera were added to each well and incubated for 1 h at 37°C, followed by horseradish peroxidase (HRP) conjugated goat anti-mouse IgG (1:5, 000 dilution, Sigma). The reaction was developed by 100 μl TMB substrate (3, 3', 5, 5'-etramethylbenzidine), and then terminated by 100 μl 2 M H_2_SO_4_. The optical densities at 450 nm were determined [38].

### Immunohistochemical staining

The cerebra and medulla of human brain tissues were from an adult and a fetus, who were both died in accidents respectively. The usage of human brain tissues and sera were permitted by Institutional Review Board (IRB) of FuYang people's hospital, where these samples were provided. For immunohistochemical staining, brain sections were deparaffinized with xylene, rehydrated in ethanol, and then treated with 0.25% trypsin solution with 0.5% CaCl_2 _in PBS for 30 min and incubated in 1% hydrogen peroxide in methanol to block endogenous peroxidase activity followed by incubation with 10% Block Ace (Sigma) in PBS. The treated sections were incubated with specific serum (1:200 dilution for patients' sera and 1:1000 dilution for mice sera with 0.05% Triton ×-100-PBS) or purified IgG fraction (1 μg/ml) from sera of EV71 infected patients at 4°C overnight. The sections were washed three times with PBS and then incubated with HRP-conjugated goat anti-human IgG (for patients' sera, 1:500 dilution, Sigma) or HRP-conjugated goat anti-mouse IgG (for innunized mice sera, 1:1000 dilution, Sigma) for 1 h at 37°C. The sections were developed with 3-3'diaminobenzidine (DAB) and examined with a light microscope [53].

### Intracranial IgG detection

IgG fraction of sera from EV71 infected patients or immunized mice were purified by Protein A conjugated agarose affinity adsorption column [54]. The mice serum was diluted in 5 volumes loading buffer: 20 mM PBS saline buffer (pH, 7.0) and loaded on a Protein A agarose affinity adsorption column. Then, after washing by 10 volumes loading buffer, the targeted IgG was eluted by 0.1 M citric acid (pH, 3.0) and dialysis against PBS buffer overnight at 4°C. 10 μg IgG were intravenous injected into neonatal mice in 2 days later after EV71 infection, and the mice were sacrificed with barbital anaesthesia in 1 days later, then the brain tissue sections were prepared to detect intracranial IgG [20]. IgG presence in brain sections were detected by immunohistochemical staining with HRP conjugated goat anti mouse secondary Ab (1:5,000, sigma) and developed with 3-3'diaminobenzidine (DAB) and examined with a light microscope.

## Competing interests

The authors declare that they have no competing interests.

## Authors' contributions

JCS and LJN conducted all experiments except for the Immunohistochemical staining and draft the manuscript. MCM, HY and XYF performed Immunohistochemical staining. LSZ, GXZ and LXL collected the EV71 infected patients sera. LWB and ZLF designed the experiment and edited the manuscript. QC provided overall supervision, financial support and prepared the final version of the manuscript. All authors read and approved the final manuscript.

## Supplementary Material

Additional file 1 **SDS-PAGE detects the purified peptides of EV71**. The SDS-PAGE result of peptides P_1-69 _to P_2072-2193 _was shown. The expression plasmids free *E. coli *BL21 (DE3) strain was manipulated as the protocol of protein expression and purification of peptides and used as negative control. The purified peptides were detected by 12% SDS-PAGE and the purified virus was detected by 8% SDS-PAGE. Click here for file

Additional file 2 **Primers used in divided polypeptides encoding regions amplification**. Click here for file

## References

[B1] Schmidt NJ Lennette EH Ho HH An apparently new enterovirus isolated from patients with disease of the central nervous system J Infect Dis 1974 129 304 309 4361245 10.1093/infdis/129.3.304

[B2] Brown BA Pallansch MA Complete nucleotide sequence of enterovirus 71 is distinct from poliovirus Virus Res 1995 39 195 205 10.1016/0168-1702(95)00087-9 8837884

[B3] Brown BA Oberste MS Alexander JP Jr Kennett ML Pallansch MA Molecular epidemiology and evolution of enterovirus 71 strains isolated from 1970 to 1998 J Virol 1999 73 9969 9975 10559310 10.1128/jvi.73.12.9969-9975.1999 PMC113047

[B4] Ortner B Huang CW Schmid D Mutz I Wewalka G Allerberger F Yang JY Huemer HP Epidemiology of enterovirus types causing neurological disease in Austria 1999-2007: detection of clusters of echovirus 30 and enterovirus 71 and analysis of prevalent genotypes J Med Virol 2009 81 317 324 10.1002/jmv.21374 19107980

[B5] Fowlkes AL Honarmand S Glaser C Yagi S Schnurr D Oberste MS Anderson L Pallansch MA Khetsuriani N Enterovirus-associated encephalitis in the California encephalitis project, 1998-2005 J Infect Dis 2008 198 1685 1691 10.1086/592988 18959496

[B6] Diedrich S Weinbrecht A Schreier E Seroprevalence and molecular epidemiology of enterovirus 71 in Germany Arch Virol 2009 154 1139 1142 10.1007/s00705-009-0413-x 19506798

[B7] Mizuta K Abiko C Murata T Matsuzaki Y Itagaki T Sanjoh K Sakamoto M Hongo S Murayama S Hayasaka K Frequent importation of enterovirus 71 from surrounding countries into the local community of Yamagata, Japan, between 1998 and 2003 J Clin Microbiol 2005 43 6171 6175 10.1128/JCM.43.12.6171-6175.2005 16333123 PMC1317214

[B8] Gau SS Chang LY Huang LM Fan TY Wu YY Lin TY Attention-deficit/hyperactivity-related symptoms among children with enterovirus 71 infection of the central nervous system Pediatrics 2008 122 e452 458 10.1542/peds.2007-3799 18606624

[B9] Lin YW Wang SW Tung YY Chen SH Enterovirus 71 Infection of Human Dendritic Cells Exp Biol Med (Maywood) 2009 19596831 10.3181/0903-RM-116

[B10] Zhu JP Xu ZG Chen H Zhang X Fan DY Wang J Primary detection of pathogen from children with hand, foot, and mouth disease in Beijing, 2007 Bing Du Xue Bao 2009 25 23 28 19437882

[B11] Ding NZ Wang XM Sun SW Song Q Li SN He CQ Appearance of mosaic enterovirus 71 in the 2008 outbreak of China Virus Res 2009 19540282 10.1016/j.virusres.2009.06.006

[B12] Lin YW Chang KC Kao CM Chang SP Tung YY Chen SH Lymphocyte and antibody responses reduce enterovirus 71 lethality in mice by decreasing tissue viral loads J Virol 2009 83 6477 6483 10.1128/JVI.00434-09 19386699 PMC2698549

[B13] Wang YF Chou CT Lei HY Liu CC Wang SM Yan JJ Su IJ Wang JR Yeh TM Chen SH Yu CK A mouse-adapted enterovirus 71 strain causes neurological disease in mice after oral infection J Virol 2004 78 7916 7924 10.1128/JVI.78.15.7916-7924.2004 15254164 PMC446098

[B14] Nishimura Y Shimojima M Tano Y Miyamura T Wakita T Shimizu H Human P-selectin glycoprotein ligand-1 is a functional receptor for enterovirus 71 Nat Med 2009 15 794 797 10.1038/nm.1961 19543284

[B15] Chen CS Yao YC Lin SC Lee YP Wang YF Wang JR Liu CC Lei HY Yu CK Retrograde axonal transport: a major transmission route of enterovirus 71 in mice J Virol 2007 81 8996 9003 10.1128/JVI.00236-07 17567704 PMC1951457

[B16] Chen YC Yu CK Wang YF Liu CC Su IJ Lei HY A murine oral enterovirus 71 infection model with central nervous system involvement J Gen Virol 2004 85 69 77 10.1099/vir.0.19423-0 14718621

[B17] Nagata N Iwasaki T Ami Y Tano Y Harashima A Suzaki Y Sato Y Hasegawa H Sata T Miyamura T Shimizu H Differential localization of neurons susceptible to enterovirus 71 and poliovirus type 1 in the central nervous system of cynomolgus monkeys after intravenous inoculation J Gen Virol 2004 85 2981 2989 10.1099/vir.0.79883-0 15448361

[B18] Nagata N Shimizu H Ami Y Tano Y Harashima A Suzaki Y Sato Y Miyamura T Sata T Iwasaki T Pyramidal and extrapyramidal involvement in experimental infection of cynomolgus monkeys with enterovirus 71 J Med Virol 2002 67 207 216 10.1002/jmv.2209 11992581

[B19] Arita M Nagata N Iwata N Ami Y Suzaki Y Mizuta K Iwasaki T Sata T Wakita T Shimizu H An attenuated strain of enterovirus 71 belonging to genotype a showed a broad spectrum of antigenicity with attenuated neurovirulence in cynomolgus monkeys J Virol 2007 81 9386 9395 10.1128/JVI.02856-06 17567701 PMC1951441

[B20] Wang H Sun J Goldstein H Human immunodeficiency virus type 1 infection increases the in vivo capacity of peripheral monocytes to cross the blood-brain barrier into the brain and the in vivo sensitivity of the blood-brain barrier to disruption by lipopolysaccharide J Virol 2008 82 7591 7600 10.1128/JVI.00768-08 18508884 PMC2493310

[B21] Bowman GL Kaye JA Moore M Waichunas D Carlson NE Quinn JF Blood-brain barrier impairment in Alzheimer disease: stability and functional significance Neurology 2007 68 1809 1814 10.1212/01.wnl.0000262031.18018.1a 17515542 PMC2668699

[B22] Ganor Y Goldberg-Stern H Amrom D Lerman-Sagie T Teichberg VI Pelled D Futerman AH Zeev BB Freilinger M Verheulpen D Autoimmune epilepsy: some epilepsy patients harbor autoantibodies to glutamate receptors and dsDNA on both sides of the blood-brain barrier, which may kill neurons and decrease in brain fluids after hemispherotomy Clin Dev Immunol 2004 11 241 252 10.1080/17402520400001736 15559370 PMC2486323

[B23] Wu Z Yang F Zhao R Zhao L Guo D Jin Q Identification of small interfering RNAs which inhibit the replication of several Enterovirus 71 strains in China J Virol Methods 2009 159 233 238 10.1016/j.jviromet.2009.04.002 19490979

[B24] Yang Y Wang H Du J Zhao XS Gong EC Gao ZF Zheng J Molecular confirmation of enterovirus type 71 infection: a post-mortem study of two cases Zhonghua Bing Li Xue Za Zhi 2009 38 258 262 19575899

[B25] Chang LY Lin TY Hsu KH Huang YC Lin KL Hsueh C Shih SR Ning HC Hwang MS Wang HS Lee CY Clinical features and risk factors of pulmonary oedema after enterovirus-71-related hand, foot, and mouth disease Lancet 1999 354 1682 1686 10.1016/S0140-6736(99)04434-7 10568570

[B26] Fujimoto T Chikahira M Yoshida S Ebira H Hasegawa A Totsuka A Nishio O Outbreak of central nervous system disease associated with hand, foot, and mouth disease in Japan during the summer of 2000: detection and molecular epidemiology of enterovirus 71 Microbiol Immunol 2002 46 621 627 12437029 10.1111/j.1348-0421.2002.tb02743.x

[B27] Ang LW Koh BK Chan KP Chua LT James L Goh KT Epidemiology and control of hand, foot and mouth disease in Singapore, 2001-2007 Ann Acad Med Singapore 2009 38 106 112 19271036

[B28] Chang LY Hsia SH Wu CT Huang YC Lin KL Fang TY Lin TY Outcome of enterovirus 71 infections with or without stage-based management: 1998 to 2002 Pediatr Infect Dis J 2004 23 327 332 10.1097/00006454-200404000-00010 15071287

[B29] Chang LY Lee CY Kao CL Fang TY Lu CY Lee PI Huang LM Hand, foot and mouth disease complicated with central nervous system involvement in Taiwan in 1980-1981 J Formos Med Assoc 2007 106 173 176 10.1016/S0929-6646(09)60236-9 17339164

[B30] Chang LY Huang LM Gau SS Wu YY Hsia SH Fan TY Lin KL Huang YC Lu CY Lin TY Neurodevelopment and cognition in children after enterovirus 71 infection N Engl J Med 2007 356 1226 1234 10.1056/NEJMoa065954 17377160

[B31] Chen CY Chang YC Huang CC Lui CC Lee KW Huang SC Acute flaccid paralysis in infants and young children with enterovirus 71 infection: MR imaging findings and clinical correlates AJNR Am J Neuroradiol 2001 22 200 205 11158910 PMC7975539

[B32] Huang CC Neurologic complications of enterovirus 71 infection in children: lessons from this Taiwan epidemic Acta Paediatr Taiwan 2001 42 5 7 11270188

[B33] Tyler KL Emerging viral infections of the central nervous system: part 1 Arch Neurol 2009 66 939 948 10.1001/archneurol.2009.153 19667214 PMC2873855

[B34] Wong KT Munisamy B Ong KC Kojima H Noriyo N Chua KB Ong BB Nagashima K The distribution of inflammation and virus in human enterovirus 71 encephalomyelitis suggests possible viral spread by neural pathways J Neuropathol Exp Neurol 2008 67 162 169 10.1097/nen.0b013e318163a990 18219253

[B35] Wu TC Wang YF Lee YP Wang JR Liu CC Wang SM Lei HY Su IJ Yu CK Immunity to avirulent enterovirus 71 and coxsackie A16 virus protects against enterovirus 71 infection in mice J Virol 2007 81 10310 10315 10.1128/JVI.00372-07 17626076 PMC2045469

[B36] Chung YC Ho MS Wu JC Chen WJ Huang JH Chou ST Hu YC Immunization with virus-like particles of enterovirus 71 elicits potent immune responses and protects mice against lethal challenge Vaccine 2008 26 1855 1862 18329759 10.1016/j.vaccine.2008.01.058

[B37] Zhou BP Liu WL Xie JJ Chen XC Xu LM Tan Y Liu YX Yang GL Expression of recombinant VP1 protein of enterovirus 71 and development of serological assay for detection of EV71 infection Zhonghua Shi Yan He Lin Chuang Bing Du Xue Za Zhi 2008 22 492 494 19544656

[B38] Chen HL Huang JY Chu TW Tsai TC Hung CM Lin CC Liu FC Wang LC Chen YJ Lin MF Chen CM Expression of VP1 protein in the milk of transgenic mice: a potential oral vaccine protects against enterovirus 71 infection Vaccine 2008 26 2882 2889 10.1016/j.vaccine.2008.03.041 18450335

[B39] Foo DG Ang RX Alonso S Chow VT Quak SH Poh CL Identification of immunodominant VP1 linear epitope of enterovirus 71 (EV71) using synthetic peptides for detecting human anti-EV71 IgG antibodies in Western blots Clin Microbiol Infect 2008 14 286 288 10.1111/j.1469-0691.2007.01904.x 18076666

[B40] Sivasamugham LA Cardosa MJ Tan WS Yusoff K Recombinant Newcastle Disease virus capsids displaying enterovirus 71 VP1 fragment induce a strong immune response in rabbits J Med Virol 2006 78 1096 1104 10.1002/jmv.20668 16789020

[B41] Chiu CH Chu C He CC Lin TY Protection of neonatal mice from lethal enterovirus 71 infection by maternal immunization with attenuated Salmonella enterica serovar Typhimurium expressing VP1 of enterovirus 71 Microbes Infect 2006 8 1671 1678 10.1016/j.micinf.2006.01.021 16815726

[B42] Chen HF Chang MH Chiang BL Jeng ST Oral immunization of mice using transgenic tomato fruit expressing VP1 protein from enterovirus 71 Vaccine 2006 24 2944 2951 10.1016/j.vaccine.2005.12.047 16448730

[B43] Olsen AL Morrey JD Smee DF Sidwell RW Correlation between breakdown of the blood-brain barrier and disease outcome of viral encephalitis in mice Antiviral Res 2007 75 104 112 10.1016/j.antiviral.2006.11.013 17223204 PMC2040264

[B44] Zanoni G Navone R Lunardi C Tridente G Bason C Sivori S Beri R Dolcino M Valletta E Corrocher R Puccetti A In celiac disease, a subset of autoantibodies against transglutaminase binds toll-like receptor 4 and induces activation of monocytes PLoS Med 2006 3 e358 10.1371/journal.pmed.0030358 16984219 PMC1569884

[B45] Bataller L Kleopa KA Wu GF Rossi JE Rosenfeld MR Dalmau J Autoimmune limbic encephalitis in 39 patients: immunophenotypes and outcomes J Neurol Neurosurg Psychiatry 2007 78 381 385 10.1136/jnnp.2006.100644 16980333 PMC2077770

[B46] Grigoriadis N Hadjigeorgiou GM Virus-mediated autoimmunity in Multiple Sclerosis J Autoimmune Dis 2006 3 1 10.1186/1740-2557-3-1 16504001 PMC1397830

[B47] Sloka S Observations on recent studies showing increased co-occurrence of autoimmune diseases J Autoimmun 2002 18 251 257 10.1006/jaut.2002.0588 12126638

[B48] Seissler J de Sonnaville JJ Morgenthaler NG Steinbrenner H Glawe D Khoo-Morgenthaler UY Lan MS Notkins AL Heine RJ Scherbaum WA Immunological heterogeneity in type I diabetes: presence of distinct autoantibody patterns in patients with acute onset and slowly progressive disease Diabetologia 1998 41 891 897 10.1007/s001250051004 9726590

[B49] Lin YC Wu CN Shih SR Ho MS Characterization of a Vero cell-adapted virulent strain of enterovirus 71 suitable for use as a vaccine candidate Vaccine 2002 20 2485 2493 10.1016/S0264-410X(02)00182-2 12057603

[B50] Lin JY Shih SR Pan M Li C Lue CF Stollar V Li ML hnRNP A1 interacts with the 5' untranslated regions of enterovirus 71 and Sindbis virus RNA and is required for viral replication J Virol 2009 83 6106 6114 10.1128/JVI.02476-08 19339352 PMC2687368

[B51] He YQ Yang H Li LL Tan J Zhou L Mao LS Yang F Liu JJ Lv X Genotype analysis of enterovirus type 71 detected from patients with hand-foot-mouth disease in Shenzhen Zhonghua Liu Xing Bing Xue Za Zhi 2008 29 790 793 19103116

[B52] Bradford MM A rapid and sensitive method for the quantitation of microgram quantities of protein utilizing the principle of protein-dye binding Anal Biochem 1976 72 248 254 10.1016/0003-2697(76)90527-3 942051

[B53] Arita M Ami Y Wakita T Shimizu H Cooperative effect of the attenuation determinants derived from poliovirus sabin 1 strain is essential for attenuation of enterovirus 71 in the NOD/SCID mouse infection model J Virol 2008 82 1787 1797 10.1128/JVI.01798-07 18057246 PMC2258712

[B54] Ito W Kurosawa Y Development of an artificial antibody system with multiple valency using an Fv fragment fused to a fragment of protein A J Biol Chem 1993 268 20668 20675 8376416

