# Supplementary figures and images for: The cross-reactivity of the enterovirus 71 to human brain tissue and identification of the cross-reactivity related fragments
Source: Virol J. 2010 Feb 22;7:47. doi: 10.1186/1743-422X-7-47 (PMC2839975; doi:10.1186/1743-422X-7-47)

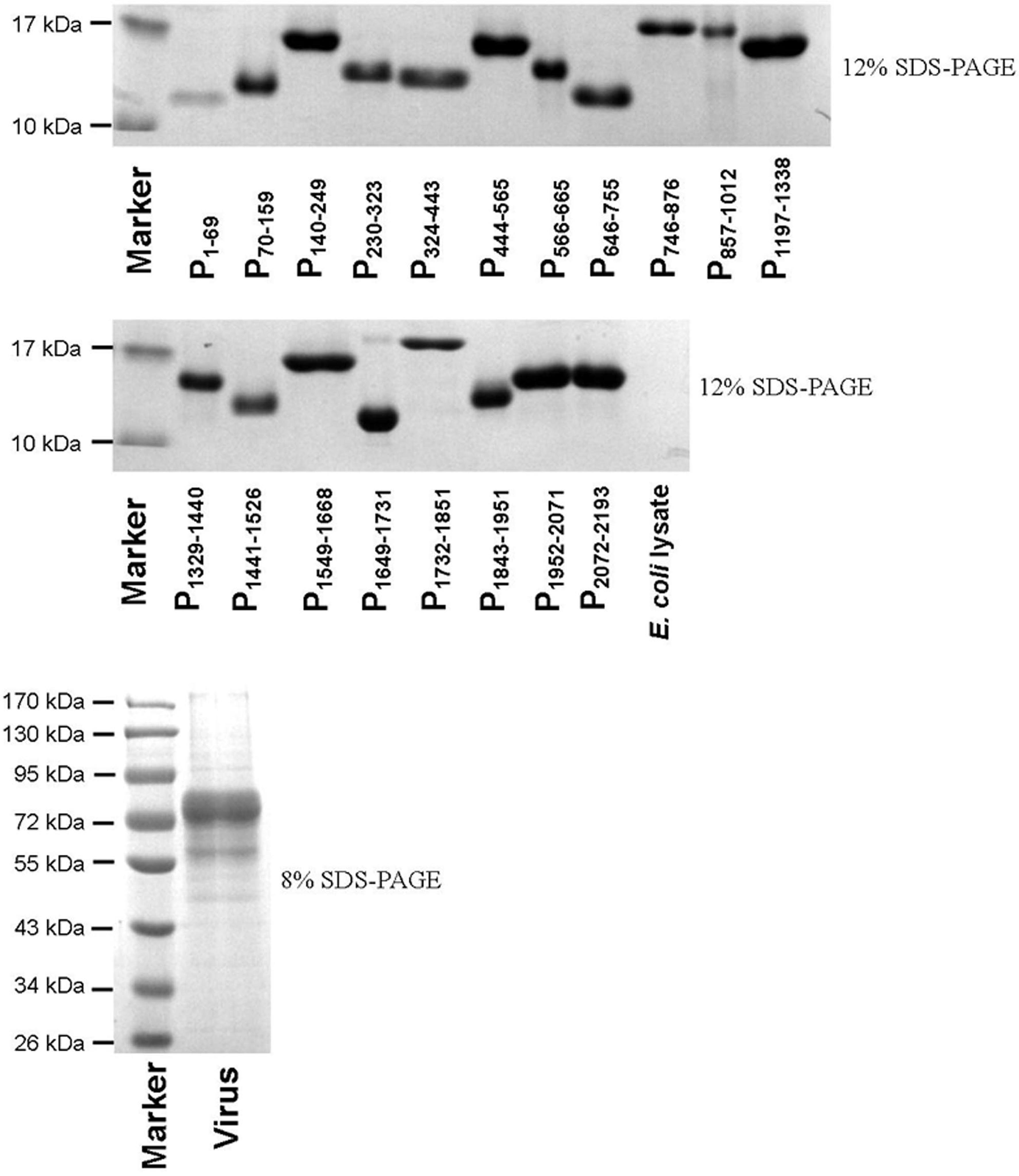

Supplement: Additional file 1 — SDS-PAGE detects the purified peptides of EV71. The SDS-PAGE result of peptides P1-69 to P2072-2193 was shown. The expression plasmids free E. coli BL21 (DE3) strain was manipulated as the protocol of protein expression and purification of peptides and used as negative control. The purified peptides were detected by 12% SDS-PAGE and the purified virus was detected by 8% SDS-PAGE. [file 1743-422X-7-47-S1.DOC]
