# Supplementary material for: The cross-reactivity of the enterovirus 71 to human brain tissue and identification of the cross-reactivity related fragments
Source: Virol J. 2010 Feb 22;7:47. doi: 10.1186/1743-422X-7-47 (PMC2839975; doi:10.1186/1743-422X-7-47)
Supplement: Additional file 2 — Primers used in divided polypeptides encoding regions amplification. [file 1743-422X-7-47-S2.DOC]

Additional file 2. Primers used in divided polypeptides encoding regions amplification.

| **Protein** | **Peptide name** | **Position in CDS** | **Primers name** | **Sequence (5’-3’)** |
| --- | --- | --- | --- | --- |
| VP4 | P_1-69_ | 1-207 | VP4-up | CATATGATGGGTTCGCAAGTGTCTACACAGCG^a^ |
|  |  |  | VP4-dw | GAGCTCTTACTTCAGTGGCGCTGCCATTTCAGTGAA |
| VP2 | P_70-159_ | 208-477 | VP2-1-up | CATATGTCCCCATCCGCTGAGGCATGTGG |
|  |  |  | VP2-1-dw | GAGCTCTTACCCAGTTTCAGTTAACACATCCGGGAAC |
|  | P_140-249_ | 418-747 | VP2-2-up | CATATGTGGGAGAAATCGTCCAAGGGATGGTACTG |
|  |  |  | VP2-2-dw | GAGCTCTTAGTGTGGGCACACTGTTAACTGTGATATTGGG |
|  | P_230-323_ | 688-969 | VP2-3-up | CATATGCAACACCCGTACGTGCTTGATGCTGG |
|  |  |  | VP2-3-dw | GAGCTCTTATTGCGTGACTGCCTGCCTAAGACCTG |
| VP3 | P_324-443_ | 970-1329 | VP3-1-up | CATATGGGGTTCCCCACCGAGCTAAAACCTG |
|  |  |  | VP3-1-dw | GAGCTCTTACATGAAGGTGACTTCCAATGATCCTGACC |
|  | P_444-565_ | 1330-1695 | VP3-2-up | CATATGTTTACTGGATCCTTCATGGCTACCGGCAAG |
|  |  |  | VP3-2-dw | GAGCTCTTACTGGATGGTGCCCGTCTGCAGGATAT |
| VP1 | P_566-665_ | 1696-1995 | VP1-1-up | CATATGGGAGATAGGGTGGCAGATGTAATTGAAAG |
|  |  |  | VP1-1-dw | GAGCTCTTATGTGCCCTTAAGAGGGAGATCTATCTCTC |
|  | P_646-755_ | 1936-2265 | VP1-2-up | CATATGGATAGTTTCTTCAGCAGGGCGGGATTAGT |
|  |  |  | VP1-2-dw | GAGCTCTTAAACCTGCGCTGGAGGGTCTGACAG |
|  | P_746-876_ | 2236-2628 | VP1-3-up | CATATGGTCAAGCTGTCAGACCCTCCAGCG |
|  |  |  | VP1-3-dw2 | GAGCTCTTAGTTGCCCACATAAATAGCCCCAGACTG |
| 2A | P_857-1012_ | 2569-3036 | 2A-1-up2 | CATATGACAGCGATCACCACTCTTGGGAAATTTGG |
|  |  |  | 2A-dw | GAGCTCTTACTGTTCCATAGCTTCTTCATCTAACCACAAG |
| 2B | P_1013-1111_ | 3037-3333 | 2B-up | CATATGGGCGTGTCCGACTACATCAAGGGTCT |
|  |  |  | 2B-dw | GAGCTCTTACTGCTTTTGAGCGATAGGGATACCTAAGATG |
| 2C | P_1112-1201_ | 3334-3603 | 2C-1-up | CATATGAGCGCTTCCTGGCTCAAGAAGTTCAATG |
|  |  |  | 2C-1-dw | GAGCTCTTAGAACTTGCGACAGAAGTGAGCTAGGTAC |
|  | P_1197-1338_ | 3589-4014 | 2C-2-up | CATATGTTCTGTCGCAAGTTCCAACCGCTATACGC |
|  |  |  | 2C-2-dw | GAGCTCTTAGATATTACTGGCATTAGTGGATGCGATGAC |
|  | P_1329-1440_ | 3985-4320 | 2C-3-up | CATATGGTCATCGCATCCACTAATGCCAGTAATATC |
|  |  |  | 2C-3-dw | GAGCTCTTATTGGAAAAGAGCCTCGATTGTGTTGCC |
| 3A | P_1441-1526_ | 4321-4578 | 3A-up | CATATGGGTCCACCCAAGTTCAGGCCAATTAGG |
|  |  |  | 3A-dw | GAGCTCTTATTGGAACCCTGCAAAGAGCTTGTAGATGAC |
| 3B | P_1527-1548_ | 4579-4644 | 3B-up | CATATGGGTGCGTATTCTGGTGCTCCTAAGCAAGT |
|  |  |  | 3B-dw | GAGCTCTTACTGTACTGTTGCTGTGCGAAGAGCAGGTTT |
| 3C | P_1549-1668_ | 4645-5004 | 3C-1-up | CATATGGGCCCGAGCCTTGACTTTGCTCTCT |
|  |  |  | 3C-1-dw | GAGCTCTTACACAACGTCACCCACCGGGACAAACATT |
|  | P_1649-1731_ | 4945-5193 | 3C-2-up | CATATGACCCTAGTGATCAACACGGAGCACATGC |
|  |  |  | 3C-2-dw | GAGCTCTTATTGTTCACTAGCAAAGTAACTCCTTTTGAGGC |
| 3D | P_1732-1851_ | 5194-5553 | 3D-1-up | CATATGGGGGAGATCCAGTGGGTTAAGCCCAA |
|  |  |  | 3D-1-dw | GAGCTCTTAATAGGGGTAACCTGCACTAGTGTGAAGATC |
|  | P_1843-1951_ | 5527-5853 | 3D-2-up | CATATGCTTCACACTAGTGCAGGTTACCCCTATAGT |
|  |  |  | 3D-2-dw | GAGCTCTTACTTGCTCCAGAATGTGTCAGGGTTACACC |
|  | P_1952-2071_ | 5854-6213 | 3D-3-up | CATATGCTGCCAATTTTGCTCCCTGGCTCACTCTT |
|  |  |  | 3D-3-dw | GAGCTCTTAATCAATTGGGAAGGGATAGCTAGCGAGCAC |
|  | P_2072-2193_ | 6214-6582 | 3D-4-up | CATATGTGCTTGGAACTAGCAAAGACTGGTAAGGAG |
|  |  |  | 3D-4-dw | GAGCTCTTAGGTGCGTGTAGCCTCTAAAAATAACTCGAG |

^a^ endonuclease sites were denoted with under line, forward primers: *Nde*I, Reverse primers: *Sac*I.
